# Feasibility and Utility of mHealth for the Remote Monitoring of Parkinson Disease: Ancillary Study of the PD_manager Randomized Controlled Trial

**DOI:** 10.2196/16414

**Published:** 2020-06-29

**Authors:** Dimitris Gatsios, Angelo Antonini, Giovanni Gentile, Andrea Marcante, Clelia Pellicano, Lucia Macchiusi, Francesca Assogna, Gianfranco Spalletta, Heather Gage, Morro Touray, Lada Timotijevic, Charo Hodgkins, Maria Chondrogiorgi, George Rigas, Dimitrios I Fotiadis, Spyridon Konitsiotis

**Affiliations:** 1 Department of Neurology Medical School University of Ioannina Ioannina Greece; 2 Unit of Medical Technology and Intelligent Information System Department of Materials Science and Engineering University of Ioannina Ioannina Greece; 3 Department of Neuroscience University of Padua Padua Italy; 4 San Camillo Hospital Istituto Di Ricovero e Cura a Carattere Scientifico Venice Italy; 5 Laboratory of Neuropsychiatry Fondazione Santa Lucia Istituto Di Ricovero e Cura a Carattere Scientifico Rome Italy; 6 Surrey Health Economics Centre Department of Clinical and Experimental Medicine University of Surrey Guildford United Kingdom; 7 School of Psychology Faculty of Health and Medical Sciences University of Surrey Guildford United Kingdom; 8 Department of Biomedical Research Institute of Molecular Biology and Biotechnology Foundation for Research and Technology-Hellas Ioannina Greece

**Keywords:** Parkinson's disease, determinants of compliance, clinically meaningful data, ecological validity

## Abstract

**Background:**

Mobile health, predominantly wearable technology and mobile apps, have been considered in Parkinson disease to provide valuable ecological data between face-to-face visits and improve monitoring of motor symptoms remotely.

**Objective:**

We explored the feasibility of using a technology-based mHealth platform comprising a smartphone in combination with a smartwatch and a pair of smart insoles, described in this study as the PD_manager system, to collect clinically meaningful data. We also explored outcomes and disease-related factors that are important determinants to establish feasibility. Finally, we further validated a tremor evaluation method with data collected while patients performed their daily activities.

**Methods:**

PD_manager trial was an open-label parallel group randomized study.The mHealth platform consists of a wristband, a pair of sensor insoles, a smartphone (with dedicated mobile Android apps) and a knowledge platform serving as the cloud backend. Compliance was assessed with statistical analysis and the factors affecting it using appropriate regression analysis. The correlation of the scores of our previous algorithm for tremor evaluation and the respective Unified Parkinson’s Disease Rating Scale estimations by clinicians were explored.

**Results:**

Of the 75 study participants, 65 (87%) completed the protocol. They used the PD_manager system for a median 11.57 (SD 3.15) days. Regression analysis suggests that the main factor associated with high use was caregivers’ burden. Motor Aspects of Experiences of Daily Living and patients’ self-rated health status also influence the system’s use. Our algorithm provided clinically meaningful data for the detection and evaluation of tremor.

**Conclusions:**

We found that PD patients, regardless of their demographics and disease characteristics, used the system for 11 to 14 days. The study further supports that mHealth can be an effective tool for the ecologically valid, passive, unobtrusive monitoring and evaluation of symptoms. Future studies will be required to demonstrate that an mHealth platform can improve disease management and care.

**Trial Registration:**

ISRCTN Registry ISRCTN17396879; http://www.isrctn.com/ISRCTN17396879

**International Registered Report Identifier (IRRID):**

RR2-10.1186/s13063-018-2767-4

## Introduction

Parkinson disease (PD) is a progressive disorder with patients having heterogeneous symptoms and progression rates. Presently, there is no cure for the condition, and treatment aims at controlling symptoms by optimizing medication plans. Optimization and personalization of the treatment is currently based on clinical interview, diaries, and scales, although in the future it may benefit from information on symptoms and medication adherence collected away from the clinic while the patients perform their normal daily activities.

Technology, even with the existing regulatory limitations and barriers, offers the possibility for improved care, self-assessment options, and overall improved health care outcomes [1]. Wearable sensors and mobile apps have been extensively used to monitor and evaluate mainly motor symptoms and motoric complications of PD patients in their home environments [2]. However, reliable and unobtrusive solutions for nonmotor symptoms are still lacking [1].

Despite the potential benefits of the use of technologies, important aspects of its feasibility remain to be explored. Only a few studies have rigorously investigated the feasibility and utility of using technology-based platforms. Moreover, apart from three studies [3-5], most prior studies remained limited by the small sample sizes (samples of up to 51 PD patients in varying disease stages) [6-11]. Evidence of mHealth utility for the clinicians is in its early days even for commercial grade systems [12,13]. None of the previous studies has systematically explored the role of caregivers in compliance with mHealth.

In this analysis, we aimed to investigate the feasibility of using an mHealth platform, described in this study as the PD_manager system, comprising a smartphone, smartwatch, and pair of smart insoles. The study focuses on participants’ compliance and their determinants. The study also validates the system’s utility to collect clinically meaningful data with ecological validity.

## Methods

### Study Population

Between May 2017 and March 2018, 136 consenting patients with PD (Hoehn and Yahr scale stage of ≥3, experiencing motor fluctuations at least 2 hours per day based on Unified Parkinson’s Disease Rating Scale [UPDRS] IV score), with a live-in caregiver, were recruited in three countries (50 in Rome and 44 in Venice, Italy; 21 in Ioannina, Greece; and 21 in Surrey, England). Four of them were excluded from the study, 2 because they withdrew and 2 because they were not eligible at reassessment, leaving a total of 75 patients assigned to the PD_manager group and 57 to the control group. The PD_manager group characteristics are summarized in Table 1.

**Table 1:** PD_manager group characteristics (n=75).

Variable	Value
Gender (female), n (%)	30 (40)
Age in years, mean (SD)	67.73 (8.72)
Years since diagnosis, mean (SD)	9.21 (4.41)
H&Y^a^ (stage 3 patients), n (%)	70 (93)
BMI (%), mean (SD)	26.04 (3.95)
MMSE^b^, mean (SD)	28.60 (1.74)
UPDRS III^c^, mean (SD)	28.15 (15.06)
NMSS^d^, mean (SD)	45.17 (38.55)

^a^H&Y: Hoehn and Yahr scale.

^b^MMSE: Mini-Mental State Examination.

^c^UPDRS III: Unified Parkinson’s Disease Rating Scale III.

^d^NMSS: Nonmotor Symptoms Scale.

In this work, we were focusing on actual system use and compliance as depicted in the data collected from the patients and their caregivers vis-à-vis data collected via the respective PD_manager devices (ie, we were analyzing only the PD_manager group). Focus was on compliance metrics as well as the factors affecting the compliance. Moreover, we provide evidence that the data are clinically meaningful since they can be used for accurately monitoring and evaluating symptoms and specifically tremor.

### Study Design

The PD_manager trial [14] was an open-label parallel group randomized study. It was conducted to assess the feasibility, usability and trends of effectiveness of the PD_manager system compared with traditional practices of using a symptom diary for the management of people with PD.

Following informed consent, baseline information was gathered, including the following: age, gender, education, attitudes toward technology (patient and caregiver), time since Parkinson diagnosis, symptom status (with Nonmotor Symptoms Scale (NMSS) and UPDRS), comorbidities (patient only), caregiver burden (Short Zarit), patient’s self-assessment of the disease (EuroQol 5-Dimension 5-Level [EQ-5D-5L]) and patient’s self-assessed quality of life (Parkinson’s Disease Questionnaire–8).

Patients were asked to use the system for 14 days continuously for 12 hours during the day. The 14-day duration for the wearing of study devices (wristband and smartphone) by participants was selected for a number of reasons. First, it was based on analysis of user needs, safeguarding ethics and privacy, as well as the burden on study participants. Second, it was considered enough for collection of sufficient data to provide clinically meaningful information. Finally, findings of previous larger studies [3], with similar investigation concepts, indicated that around 70% of the patients were compliant for up to 15 days.

During the 14-day period, the system passively and automatically captured raw sensor data (from the smartphone, wristband, and insoles) to be used for the evaluation of motor symptoms, aggregated data on sleep and activity (wristband proprietary software), speech, cognitive status, and emotional state using the smartphone apps (with scheduled prompts for the user to perform specific tasks). The smartphone was used for storing the data locally. Automatic transmission of the data to a cloud backend was possible but not used during the pilot for privacy and security purposes. Control group participants were asked to keep a motor symptom diary for 3 days and complete the Parkinson Well-Being Map. After a minimum of 2 weeks, a specialist doctor reviewed the data gathered. Participants, caregivers, and clinicians were asked for feedback on the acceptability and utility of the data collection methods. Data collection for the pilot study is summarized in Table 2.

**Table 2:** Summary of PD_manager group data collection at each stage.

Participant group	Data capture at each stage		
	Baseline	During intervention, PD_manager group from devices	Postintervention, 2-week follow-up
Patient	Age, gender, education, disease duration, disease stage (Hoehn and Yahr score), main symptoms (tremor, bradykinesia, rigidity, dyskinesia), more affected side, UPDRS^a^ scores, current medications, comorbidities, views on technology (with the TAMM^b^). Outcomes: EQ-5D-5L^c^, PDQ-8^d^, NMSS^e^, UPDRS	Motor symptoms (gait, freezing of gait, bradykinesia, dyskinesia, activity); nonmotor symptoms (cognition, sleep, mood)	Interviews on acceptability and ease of use of PD_manager or symptom diary. Data collected in the smartphone and in the backend from smartphone and wristband sensors, data from insoles stored in the backend
Caregiver	Age, gender, education, views on technology (with the TAMM). Outcome: Zarit Caregiver Burden Scale (short version)	No information was collected from caregivers in the PD_manager group	Interviews on acceptability and ease of use of PD_manager or symptom diary
Clinician	Technophobia, previous experience with monitoring technology, sociodemographics, clinical experience	—	SUS^f^, PSSUQ^g^, TAMM.

^a^UPDRS: Unified Parkinson’s Disease Rating Scale.

^b^TAMM: technology acceptance modified model.

^c^EQ-5D-5L: EuroQol 5-Dimension 5-Level.

^d^PDQ-8: Parkinson’s Disease Questionnaire–8.

^e^NMSS: Nonmotor Symptoms Scale.

^f^SUS: system usability scale.

^g^PSSUQ: Poststudy System Usability Questionnaire.

### mHealth Platform

**Figure 1:**
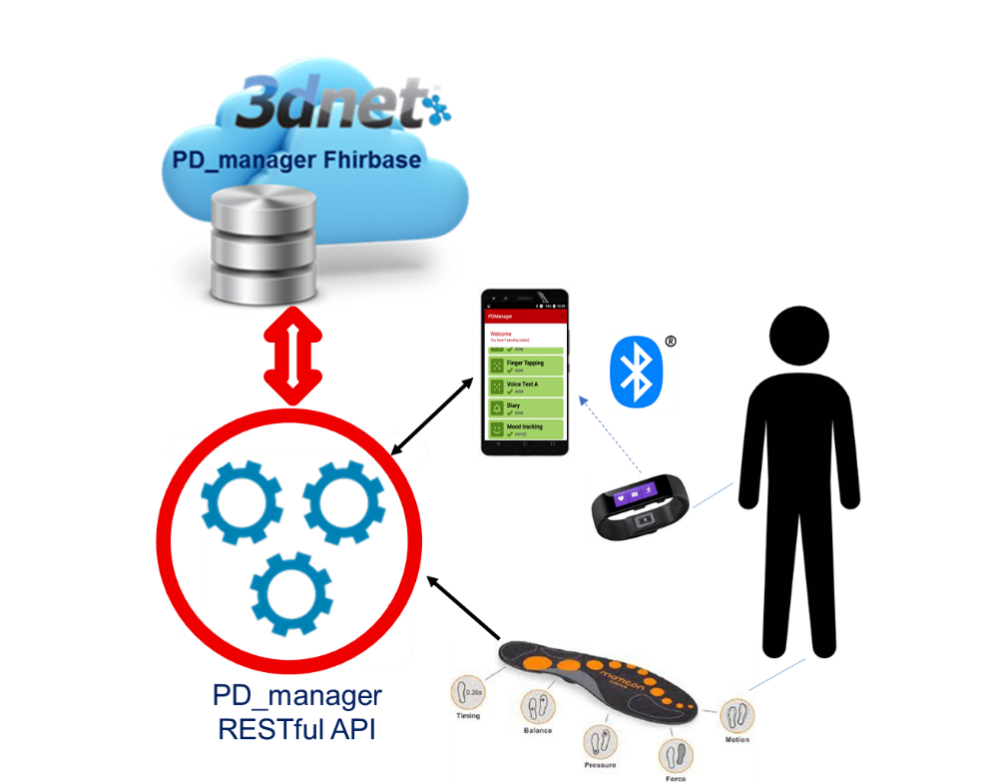
PD_manager mHealth platform overview.

**Figure 2:**
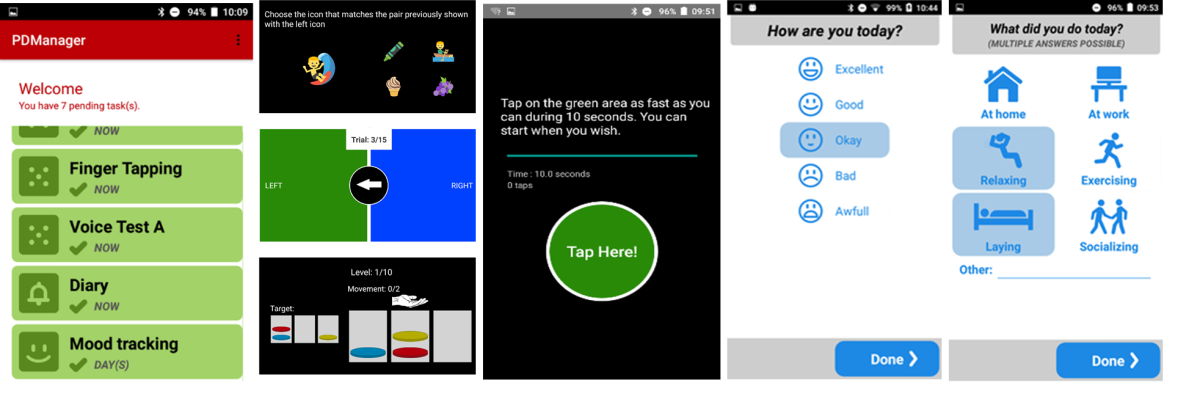
Screenshots from mobile app. From left to right: tasks patient must perform, some cognitive tests, finger-tapping test, mood monitoring diary.

The mHealth platform depicted in Figure 1 has been described in detail in a previous work [15] and consists of a wristband (Microsoft Band, Microsoft Corporation), a pair of sensor insoles (Moticon GmbH), a smartphone (Aquaris M and U models, BQ) with dedicated mobile Android apps (see Figure 2) and a knowledge platform (hosted by Biotronics 3D) serving as the cloud backend of the platform. The Microsoft Band software development kit allowed us to access data from the Band’s sensors. The wristband and smartphone provided raw data from the 3-axis accelerometer and gyroscope at a sampling rate of 100 Hz that were used for building motor symptoms’ assessment methods. The Band could also be used for collecting heart rate, galvanic skin response, and skin temperature data. Moreover, the accompanying Microsoft Health App provided aggregated data for sleep (sleep duration, number of wakeups, ratio of time asleep to total sleep, total length of restless, and restful sleep in minutes) and activity type (eg, run, sleep, bike, summary of calories burned, summary of heart rate data). With the insoles, we collected pressure distribution and accelerometer data enabling us to evaluate weight-bearing, balance and motion sequences, and study gait.

Data from devices were transferred and stored in a web-based cloud, NoSQL database in anonymized and encrypted format. The servers storing the information in the cloud platform are based on Biotronics 3D’s 3DnetMedical platform in an ISO 27001-accredited data center located in London. They are operated in accordance with the Data Protection Act.

During the pilot study, participants were instructed to always carry the smartphone with them since the wristband needs to be paired with the phone through the Bluetooth connection for transmitting and storing wristband data. Two optimization strategies were applied in order to reach the desired Microsoft Band battery daily duration of 12 to 14 hours and address battery drain issues: (1) by default, the app acquires data for a period of 5 minutes and then disconnects from Microsoft Band (closing Bluetooth and therefore significantly reducing the Microsoft Band power consumption) for a period of X minutes, where X is estimated based on the hours of the required recording interval which is customized in the app settings, and (2) when the patient removes the Microsoft Band (detected with the heart rate quality value), the data acquisition is postponed. Moreover, study participants were instructed to use the system as much as possible during the waking day while performing daily activities and charge it just before going to sleep. The insoles had their internal storage capability.

The devices are unobtrusive. Their wearability, sensitivity, and reliability were tested as part of an earlier proof of concept study [16] with 20 patients (5 Rome and 10 Venice, Italy; 5 Ioannina, Greece). This proof-of-concept study was supervised by neurologists in an in-hospital setting and involved short sessions (154 in total, each lasting around 30 minutes) following a common protocol that included simulation of daily activities such as opening a door, drinking water, walking a few meters, rising from a chair, and rising from the bed. The nutrition and physiotherapy modules were evaluated in separate studies [17].

**Figure 3:**
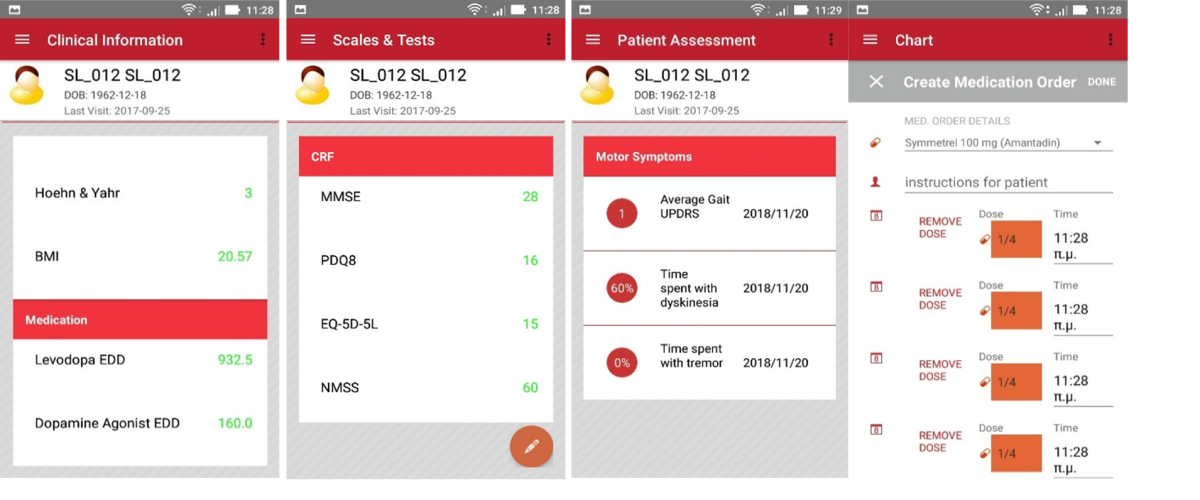
Screenshots from clinician mobile app. From left to right: overview of clinical information, scores from scales and tests, overview of motor symptoms as assessed by the PD_manager, and create new medication order. To ensure there are no risks for participants, we omitted the medication adherence module (mobile app and pillbox) from pilot.

The clinicians had a dedicated mobile app (see Figure 3) that enabled them to check the demographic and clinical information, assess the overall status of the patient, evaluate symptoms monitored during the pilot period, and get decision support functionalities [18] on patient mobility.

### Outcome Definitions and Statistical Analysis

Feasibility assessment included recruitment, compliance, and evaluation of the processed sensor data utility for answering clinically meaningful questions. Recruitment success was analyzed by the total number of enrolled, consenting participants who completed the pilot study against dropouts. Compliance was calculated as the total hours where band and smartphone sensor data were collected during the 14-day period, as well as number of days during which the participant used the system for at least 1 hour.

The statistical analysis investigated the effects of the patient demographics (age, gender, education), clinical symptoms (as depicted in the Nonmotor Symptom Scale and UPDRS), self-rated quality of life (Parkinson’s Disease Questionnaire–8 (PDQ-8) and EQ-5D-5L), caregivers’ demographics (age, gender, education), and burden (as captured with the short version of Zarit) on the system use as reflected in the total use hours over the 14-day data collection period by the devices for each participant. In this targeted analysis, we have included only the 65 of the originally recruited 75 participants for which duration of data collected is at least one day of the pilot period. The study data were analyzed by SPSS Statistics version 23 software (IBM Corporation).

Compliance was not normally distributed. Correlations between compliance and the available at-baseline information for the participants were explored with Spearman rank-order and Kendall tau-b. Participants were then divided in low, moderate, and high use groups using the quartiles (the first quartile was the cutoff for the low compliant group and third quartile for the high compliant group) and taking into account qualitative information, mainly Band use—another metric available for compliance evaluation—for confirming the grouping. Significant differences in the distributions of use between compliance groups were investigated with a Kruskal-Wallis H test for the low, moderate, and high groups.

To further investigate factors affecting compliance, regression analysis was applied. Linear regression determined how much of the variation in the use was explained by the caregiver burden. Multiple linear regression determined how much of the variation in the system use was explained by the caregivers’ burden, Motor Aspects of Experiences of Daily Living, and patient self-rated health statuses. Binary logistic regression explored the effects of the same parameters on the likelihood of use, predicting the moderate and high groups.

**Figure 4:**
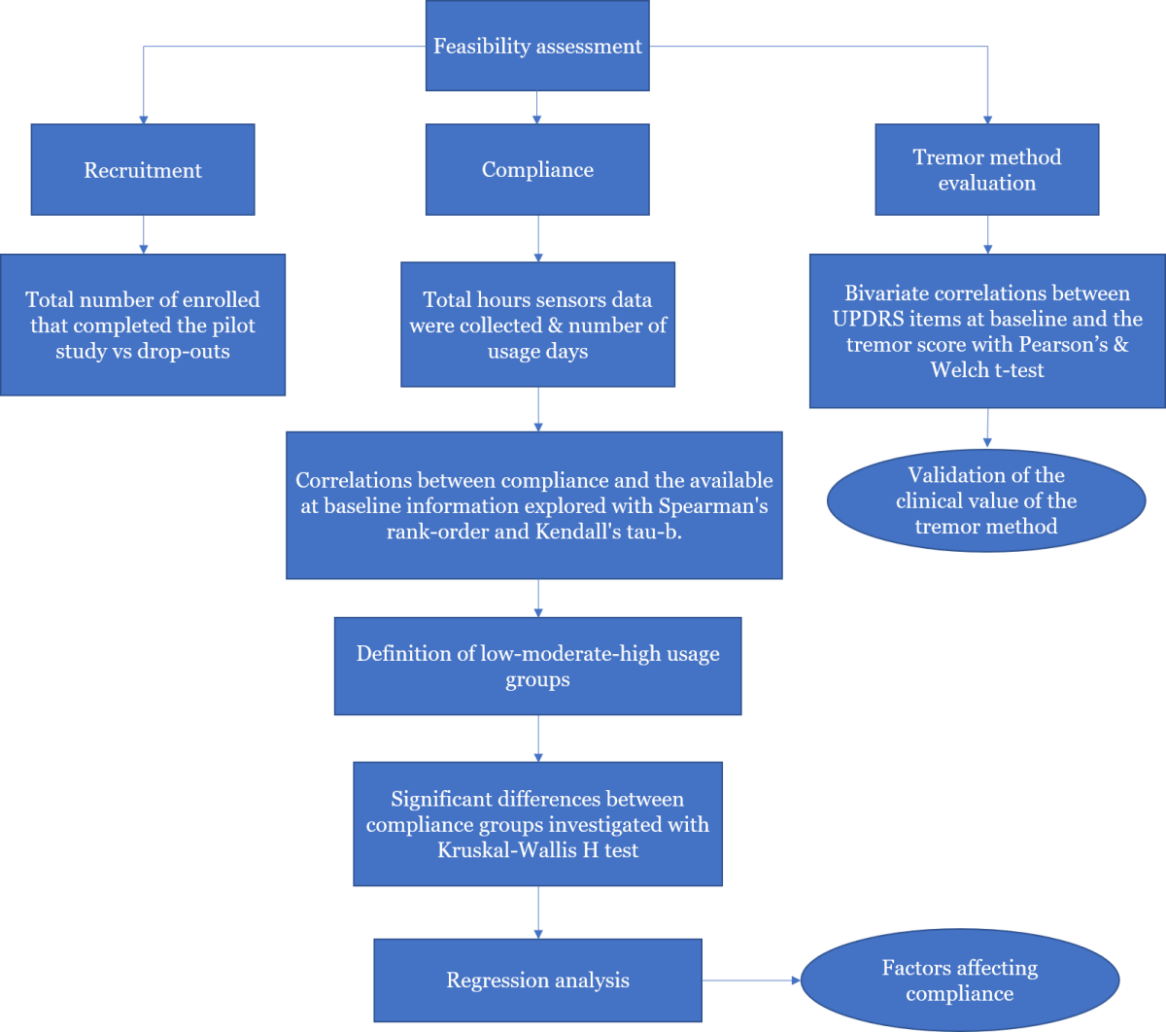
Outcome definitions and statistical analysis.

Validation of the tremor method was done with bivariate correlations (with Pearson test) between UPDRS items scored by the clinicians at baseline, and the tremor score calculated with our method. A Welch *t* test was also run to determine if there were differences in scores between the no-tremor and tremor groups. The statistical methods used in the analysis are depicted in Figure 4.

## Results

### Recruitment

From the 75 patients who were eligible and consented to participate in the study and were randomly assigned to the PD_manager group, 65 (87%) were data providers with at least 1 day of system use. The other 10 either chose not to use the system or due to technical reasons (Bluetooth disconnection) were unable to use it.

### Compliance

The 65 data contributors collected data for a median of 63.37 (SD 42.17) hours total in the 14-day study period (ie, 4.53 hours on average per day). They used the system for a median 11.57 (SD 3.15) days. Only two of the study participants used the system for 1 day. All others used it for more than 6 days, with 30 using it for the whole 14-day study period.

### Sample Characteristics and Bivariate Correlations

Study sample characteristics are presented in Table 3. Most participants had many symptoms as reflected in UPDRS total and subscores. Most study participants were men (almost 2:1), while for caregivers the reverse was observed (women 2:1). Caregivers were slightly more educated (11.96 years) compared with study participants (10.18 years), which can be explained by the fact that 27% were children or nephews. For the same reason, the caregivers were younger (mean 60 years). UPDRS score (mean 56.45) is consistent with the severity of the condition. Participants did not have dementia (based on Mini-Mental State Examination and instrumental activities of daily life).

**Table 3:** Analysis of distributions between groups.

Characteristic	High group (n=21)		Moderate group (n=28)		Low group (n=16)			χ^2^_2_ (*P* value)	
	Mean (SD)	Correlation coefficient, *P* value (spearman)	Mean (SD)	Correlation coefficient, *P* value (spearman)		Mean (SD)	Correlation coefficient, *P* value (spearman)		
Use hours	108.29 (37.50)		58.93 (18.47)			19.19 (7.63)			
Days of use	13.52 (1.03)	.10 (.66)	11.21 (2.59)	–.03 (.88)		9.63 (4.40)	.34 (.20)		14.8 (.001)
Caregiver age	60.00 (13.09)	.19 (.43)	58.38 (12.06)	.17 (.40)		63.50 (10.83)	–.15 (.64)		2.1 (.34)
Caregiver education	10.89 (4.90)	–.26 (.28)	12.50 (5.02)	.13 (.54)		12.60 (4.20)	.09 (.80)		0.7 (.71)
Patient age	67.24 (6.71)	–.23 (.31)	67.67 (11.45)	.19 (.35)		67.69 (6.02)	–.46 (.08)		0.1 (.95)
Patient education	9.50 (4.87)	–.42 (.07)	10.48 (4.23)	.002 (.99)		10.50 (5.07)	.07 (.80)		0.3 (.87)
Disease duration	8.90 (5.04)	.04 (.85)	9.18 (4.67)	–.08 (.69)		8.44 (3.08)	–.34 (.20)		0.005 (>.99)
MMSE^a^	28.32 (1.70)	.58 (.01)	28.79 (2.13)	.17 (.38)		28.40 (1.24)	–.26 (.34)		0.9 (.65)
EQ-5D-5L^b^ total	9.48 (3.63)	–.20 (.38)	10.54 (3.42)	.06 (.78)		9.87 (2.70)	–.13 (.64)		1.0 (.61)
NMSS^c^ total	42.09 (29.43)	–.16 (.48)	44.00 (34.85)	.21 (.29)		39.07 (17.62)	.30 (.31)		0.2 (.91)
PDQ-8^d^ total	41.25 (18.41)	–.10 (.68)	43.29 (23.95)	.19 (.34)		39.58 (17.30)	.32 (.24)		0.2 (.91)
UPDRS^e^ I total	10.81 (6.65)	–.05 (.82)	11.11 (5.42)	.12 (.55)		11.00 (4.40)	–.17 (.56)		0.3 (.87)
UPDRS II total	13.14 (9.14)	–.04 (.87)	10.54 (7.39)	.35 (.07)		8.36 (6.01)	–.31 (.28)		2.4 (.30)
UPDRS III total	29.67 (17.46)	–.08 (.73)	28.85 (15.28)	.20 (.32)		25.67 (13.93)	.08 (.77)		0.5 (.76)
UPDRS IV total	5.76 (3.90)	.23 (.31)	5.93 (4.60)	.07 (.71)		5.58 (3.44)	.44 (.12)		0.1 (.97)
UPDRS total	59.38 (30.74)	.02 (.92)	56.70 (28.10)	.21 (.29)		51.57 (24.67)	–.08 (.80)		0.7 (.71)
Zarit total	14.67 (9.90)	.28 (.21)	8.92 (6.93)	.21 (.31)		10.64 (7.22)	–.20 (.49)		4.3 (.12)
EQ-5D-5L item 4 (pain/discomfort)	1.81 (0.87)	–.07 (.76)	2.64 (0.95)	–.11 (.58)		2.33 (0.90)	.11 (.69)		8.5 (.01)
NMSS item 11 (flat moods)	1.33 (1.96)	.11 (.65)	1.07 (2.22)	.18 (.37)		0.07 (0.27)	–.07 (.82)		7.4 (.03)
NMSS item 26 (problems having sex)	2.24 (4.19)	–.31 (.17)	0.82 (3.14)	.12 (.57)		0.36 (1.08)	.09 (.75)		6.2 (.045)
PDQ-8 item 7 (painful cramps or spasms)	1.70 (1.22)	–.005 (.99)	2.59 (1.48)	.03 (.90)		2.27 (1.23)	.51 (.52)		6.2 (.046)
UPDRS item 21 (speech)	1.43 (1.03)	–.09 (.69)	0.75 (0.93)	.14 (.48)		0.57 (0.76)	.13 (.65)		8.4 (.02)
UPDRS item 33a (rigidity)	1.19 (0.98)	.36 (.11)	0.54 (0.64)	.08 (.71)		0.73 (0.80)	–.04 (.90)		6.5 (.04)
Zarit item 8 (social life suffered)	1.29 (1.19)	.24 (.30)	0.62 (0.80)	.16 (.44)		0.43 (0.76)	–.13 (.66)		7.3 (.03)

^a^MMSE: Mini-Mental State Examination.

^b^EQ-5D-5L: EuroQol 5-Dimension 5-Level.

^c^NMSS: Nonmotor Symptoms Scale.

^d^PDQ-8: Parkinson’s Disease Questionnaire–8.

^e^UPDRS: Unified Parkinson’s Disease Rating Scale.

### Determinants of Compliance

A Kruskal-Wallis H test was run to determine if there were differences in use between patients’ groups (low, moderate, and high use) based on demographics and total scores as well as on their scoring in the ordinal variables which are indicating symptoms (NMSS and UPDRS items), quality of life aspects (PDQ-8 and EQ-5D-5L items) and caregiver burden reasons (Zarit items). The distributions of use were significantly different between groups for specific items of the scales and not for the total scores (Table 3).

A linear regression was run to understand the effect of caregivers’ burden on system use. Linearity was assessed with a scatterplot of Zarit_total against system use in which the regression line was plotted. Visual inspection of these two plots indicated a linear relationship between the variables. There was homoscedasticity and normality of the residuals. There were no outliers.

The prediction equation was: use = 48.31 + 1.51 * Zarit_total. Zarit_total statistically significantly predicted use, *F*_1,59_=5.86, *P*<.02, accounting for 30% of the variation in use with adjusted *R*^2^=7.5%, a small size effect according to Cohen.

A multiple regression analysis was run to determine how much of the variation in the system use can be explained by the caregivers’ burden (Zarit total), Motor Aspects of Experiences of Daily Living (UPDRS II), and patients self-rated health status (EQ-5D-5L).

There was linearity as assessed by partial regression plots and a plot of studentized residuals against the predicted values. There was independence of residuals, as assessed by a Durbin-Watson statistic of 1.855, and homoscedasticity, as assessed by visual inspection of a plot of studentized residuals versus unstandardized predicted values. There was no evidence of multicollinearity, as assessed by tolerance values greater than 0.1. There were no studentized deleted residuals greater than 3 standard deviations and values for the Cook distance above 1 as well as leverage values greater than 0.2 (outliers). The assumption of normality was met, as assessed by a Q-Q plot. The multiple regression model statistically significantly predicted use, *F*_3,56_=5.650, *P*=.002. *R* for the overall model was 48.2% with an adjusted *R*^2^ of 19.1%, a medium size effect according to Cohen. All three variables added statistically significantly to the prediction, *P*<.05. Regression coefficients and standard errors can be found in Table 4.

**Table 4:** Summary of multiple regression analysis.

Model	B^a^	SE_B_^b^	Beta^c^	*P* value
Constant	89.084	16.345	—	<.001
EQ-5D-5L^d^ total	–6.022	1.925	–0.465	.003
Zarit total	1.651	0.687	0.331	.02
UPDRS^e^ II total	1.757	0.847	0.326	.04

^a^B: unstandardized regression coefficient.

^b^SE_B_: SE of the coefficient.

^c^Beta: standardized coefficient.

^d^EQ-5D-5L: EuroQol 5-Dimension 5-Level.

^e^UPDRS: Unified Parkinson’s Disease Rating Scale.

A binomial logistic regression was performed to ascertain the effects of caregivers’ burden (Zarit total), Motor Aspects of Experiences of Daily Living (UPDRS II), and patients’ self-rated health status (EQ-5D-5L) on the likelihood of high system use. Linearity of the continuous variables with respect to the logit of the dependent variable was assessed via the Box-Tidwell procedure. A Bonferroni correction was applied using all 8 terms in the model resulting in statistical significance being accepted when *P*<.008. Based on this assessment, all continuous independent variables were found to be linearly related to the logit of the dependent variable. There was one standardized residual. The logistic regression model was statistically significant (χ^2^_3_=13.5, *P*=.004). The model explained 33.3% (Nagelkerke *R*^2^) of the variance in use and correctly classified 74.5% of cases. Sensitivity was 80.8%, specificity was 66.7%, positive predictive value was 75.0%, and negative predictive value was 73.3%. Of the three predictor variables, two were statistically significant: caregivers’ burden and patients’ self-rated health status as shown in Table 5. Users with better self-rated health status had 1.5 times higher odds to exhibit higher system use. Moreover, increasing caregivers’ burden was associated with an increased likelihood of higher system use.

**Table 5:** Summary of binary logistic regression.

Model	B^a^	SE_B_^b^	Wald χ^2^_1_	*P* value	Odds ratio
EQ-5D-5L^c^ total	0.396	0.158	6.252	.01	1.485
UPDRS^d^ II total	–0.088	0.057	2.395	.12	0.916
Zarit total	–0.122	0.053	5.186	.02	0.885
Constant	–1.294	1.119	1.338	.25	0.274

^a^B: unstandardized regression coefficient.

^b^SE_B_: SE of the coefficient.

^c^EQ-5D-5L: EuroQol 5-Dimension 5-Level.

^d^UPDRS: Unified Parkinson’s Disease Rating Scale.

### Clinically Meaningful Data With Ecological Validity

The method for the evaluation of tremor was presented in Rigas et al [19]. The limitation of this method was that the validation of accuracy was done with annotations by clinicians over specific, short periods in the controlled environment of a clinic, following a specific protocol [16]. In this first data collection study, the sessions were filmed in order to validate the annotations with external observers. With the data collected in the pilot study presented here, we were able to evaluate whether the method works for patients performing daily activities. Video at home was excluded due to study participants’ privacy concerns. The annotation was the perceived tremor as depicted in UPDRS item 2.10 (which indicates how the patient experienced tremor over the past week), the rest tremor amplitude in the left and right upper extremity as depicted in UPDRS item 3.17 (which allows the rater to gather observations on rest tremor that may appear at any time during the exam), and the constancy of rest tremor as depicted in UPDRS item 3.18 (which focuses on the constancy of rest tremor during the examination period when different body parts are variously at rest). All UPDRS items were assessed at the baseline visit (ie, before the pilot use of the system). Maximum of 3.17a and 3.17b referring to rest tremor amplitude in upper extremities was also estimated as part of the analysis.

A total of 50 cases were included in tremor analysis since for these cases more than 30 hours of sensor data were available from the pilot study and the results can be considered as reliable. The tremor was constantly evaluated at any moment data were available from the system. For the evaluation the method presented in Rigas et al [19] was used, and this is the score depicted in Table 6. Bivariate correlations between UPDRS items scored by the clinicians at baseline and the tremor score with our method were calculated with the Pearson product-moment correlation coefficient.

We notice that the mean score for no tremor is close to zero. This is due to the fact that some daily movements can simulate tremor and, as explained, the score was constantly calculated. Consistently, we noticed a small increase of mean score for slight tremor and a more significant increase for mild and moderate tremor.

A Welch *t* test was run to determine if there were differences in scores between groups and statistically significant differences confirm the discrimination between the no-tremor and tremor groups.

Moreover, there is a statistically significant, strong positive correlation between the tremor score and amplitude and constancy of tremor as evaluated at baseline by the clinicians and a moderate positive correlation with tremor as perceived by the patient.

**Table 6:** Correlations between Unified Parkinson’s Disease Rating Scale tremor-related items and our tremor method scores.

UPDRS^a^ item	2.10 (tremor as perceived by the patient)	3.17-a (rest tremor amplitude—right upper extremity)	3.17-b (rest tremor amplitude—left upper extremity)	Max 3.17
Cases with UPDRS=0, n	21	39	38	32
Scores for UPDRS=0, mean (SD)	0.038 (0.034)	0.073 (0.156)	0.066 (0.116)	0.037 (0.032)
Cases with UPDRS=1, n	18	8	8	12
Scores for UPDRS=1, mean (SD)	0.123 (0.226)	0.184 (0.248)	0.220 (0.329)	0.128 (0.172)
Cases with UPDRS>1, n	11	3	4	6
Scores for UPDRS>1, mean (SD)	0.267 (0.330)	0.538 (0.383)	0.421 (0.383)	0.540 (0.377)
Area under the curve	0.643	0.887	0.783	0.871
Welch *t* test, *P* value for UPDRS=0 and UPDRS>1	0.005	<0.001	<0.001	<0.001
Welch *t* test, *P* value for UPDRS=0 and UPDRS=1	0.108	0.117	0.03	0.008
Pearson correlation	.378	.544	.468	.711
Pearson *P* value	.007	<.001	<.001	<.001

^a^UPDRS: Unified Parkinson’s Disease Rating Scale.

## Discussion

### Principal Findings

The most important finding of our study is that patients with moderate PD, regardless of their age, gender, education, severity of symptoms, specific symptoms, perceived quality of life, or caregiver burden, were compliant with the use the system for 1 to 2 weeks. Overall, 87% (65/75) of study participants were data contributors for 4.53 hours on average per day. They used the system for a median 11.57 (SD 3.15) days.

Regarding the compliance determinants, regression analysis suggests that the best predictor associated with system use was caregiver burden. The higher the burden the higher the use, a finding emphasizing the role of caregivers in adherence to mHealth solutions including wearables. Moreover, deterioration of a caregiver’s social life seemed to be the most influential factor among Zarit items. The implication of these findings is that the moderate use group demonstrated the lower caregiver burden.

Motor Aspects of Experiences of Daily Living (UPDRS II) also affect the use of the system with users facing several motor problems in their activities of daily life belonging to the high use group and the rest decreasingly in the moderate and low groups. Speech problems especially seem to discriminate groups. Moreover, patients self-rated health status seems to predict high and moderate use. Feeling pain or discomfort was the strongest individual predictor.

Another objective of the study was to collect data that are clinically meaningful (ie, data that the clinicians can use for monitoring and evaluation of symptoms when the patient is in his or her home environment. In this study, we provide evidence of clinical validity and ecological effect of an algorithm derived from a single sensor on the wrist for detecting tremor in PD patients. The applications of such monitoring methods include patients who cannot properly report their symptoms either because they are newly diagnosed or because they find it difficult to characterize tremor or even differentiate tremor from dyskinesias.

Following the paradigm of recent studies, PD_manager has built a large database for future development and testing of novel algorithms applied to sensor-derived data from PD patients during daily functioning. In total, more than 2700 hours of useful sensor data from the smartphone and Microsoft Band were collected and can be used for evaluating gait, freezing of gait, bradykinesia, tremor and dyskinesia, or monitoring and evaluation of fluctuations in future studies.

### Comparison With Prior Work

This study contributes to the growing evidence about the feasibility of mHealth for PD patients. It is aligned with the findings that there are no noteworthy variances in baseline characteristics (age, gender, education, disease duration, and severity) that can explain compliance even in larger studies [4]. Findings such as highest compliance of older participants in one study [9], which can be attributed to more severe disease status and increased need for better management, and a negative impact of patients’ and caregivers’ education in this study, which can be the result of the lack of direct feedback from the system leading to limited self-management value, are worthy of further exploration.

By including patients with moderate disease severity (H&Y of 3) and by exploring the determinants of their compliance, PD_manager complements most prior studies that recruited mostly patients mildly affected (H&Y of 2 or less) [4,9-11,13]. The high level of system use and compliance of these more affected patients, as in previous studies, can be linked to factors including the simple and passive design of the patient’s app, which was basically providing a series of reminders for short motor and nonmotor tasks, the insight in the condition that the patients and their caregivers expect as a result of using the system, and the fact that the technology is considered an extension of prescribing clinicians and thus very important for better care. PD_manager was used as a PD Holter (ie, in a similar context as sensors used in recent studies [8,10,13]), and this complements the findings from previous research [3-5,9] that suggests that mHealth systems could be used both for short (1-2 weeks) and long-term (6 months) monitoring of PD patients.

Moreover, our findings are consistent with the Movement Disorder Society Task Force on Technology roadmap [20] as well as with patient attitudes on technology use [21]. Our mHealth platform, as relevant studies suggest, can be an effective tool for the passive, unobtrusive monitoring and evaluation of symptoms [22], defining new phenotypical biomarkers [23], detection of serious events such as falls [24], detection of worsening in the overall health status of the patients, and the provision of better disease management and improved care [25], the latter being already extensively studied in ongoing clinical trials (eg, NCT03741920 and NCT02657655). mHealth may also help rehabilitation [26,27] and facilitate telemedicine since it enables home-based [28], multidisciplinary [29] approaches for the management of PD. Moreover, the system could be used for connecting and sharing health data promoting research in PD [30] in line with EU priorities for enabling the digital transformation of health and care. Empowering citizens and promoting self-management is another important benefit of mHealth for PD patients [5]. Finally, mHealth can be used to provide decision support on the need for advanced treatments and their titration when they are applied [31].

### Limitations

Limitations include the number of patients that used the system which, despite the excellent stratification that was preferred in this study over extended recruitment, should be increased in future studies to further establish the findings. The relatively preserved cognitive condition of study patients could be considered a limitation since cognitive deficits are common in advanced PD. Compliance was not calculated as the median percentage of the study period where accelerometer data were collected as in previous studies because the designs are different, and the technology limitations imposed a rather personalized use of the system during the waking day. Another limitation is that compliance should also be assessed in repeated 10- to 14-day periods at least twice a year as clinically meaningful use would demand to evaluate the long-term effects in patients’ care. Finally, more workshops with clinicians for improving the use of the system in clinical practice are required.

### Conclusions

mHealth for monitoring of PD patients’ symptoms is feasible, at least for a period of 2 weeks. With the data collected with mHealth, ecologically valid, accurate, and objective monitoring and evaluation of tremor and other symptoms is feasible, and future studies should confirm its efficiency to support clinical decisions and improve patients’ management. Future mHealth systems should take into consideration and address the determinants of mHealth use, which include the subjective caregiver burden and especially the impact on social life, the self-evaluation of the activities of daily life including speech, and the overall patients’ self-rated health status with emphasis in pain and discomfort.

## Abbreviations

EQ-5D-5L: EuroQol 5-Dimension 5-Level
H&Y: Hoehn and Yahr scale
NMSS: Non-motor symptoms scale
PD: Parkinson disease
PDQ-8: Parkinson's Disease Questionnaire-8
UPDRS: Unified Parkinson’s Disease Rating Scale
